# Identifying major depressive disorder in older adults through naturalistic driving behaviors and machine learning

**DOI:** 10.1038/s41746-025-01500-w

**Published:** 2025-02-15

**Authors:** Chen Chen, David C. Brown, Noor Al-Hammadi, Sayeh Bayat, Anne Dickerson, Brenda Vrkljan, Matthew Blake, Yiqi Zhu, Jean-Francois Trani, Eric J. Lenze, David B. Carr, Ganesh M. Babulal

**Affiliations:** 1 Department of Neurology, Washington University School of Medicine, St. Louis, MO USA; 2 Department of Biomedical Engineering, Schuluch School of Engineering, University of Calgary, Calgary, AB Canada; 3 Department of Geomatics Engineering, Schuluch School of Engineering, University of Calgary, Calgary, AB Canada; 4 Department of Occupational Therapy, East Carolina University, Greenville, NC USA; 5 Occupational Therapy, School of Rehabilitation Science, McMaster University, Hamilton, ON Canada; 6 Brown School, Washington University in St. Louis, St. Louis, MO USA; 7 Institute of Public Health, Washington University in St. Louis, St. Louis, MO USA; 8 National Conservatory of Arts and Crafts, Paris, France; 9 Centre for Social Development in Africa, Faculty of Humanities, University of Johannesburg, Johannesburg, South Africa; 10 Department of Psychiatry, Washington University School of Medicine, St. Louis, MO USA; 11 Department of Medicine, Washington University School of Medicine, St. Louis, MO USA

**Keywords:** Dementia, Depression, Machine learning, Predictive medicine, Data mining

## Abstract

Depression in older adults is often underdiagnosed and has been linked to adverse outcomes, including motor vehicle crashes. With a growing population of older drivers in the United States, innovations in screening methods are needed to identify older adults at greatest risk of decline. This study used machine learning techniques to analyze real-world naturalistic driving data to identify depression status in older adults and examined whether specific demographics and medications improved model performance. We analyzed two years of GPS data from 157 older adults, including 81 with major depressive disorder, using XGBoost and logistic regression models. The top-performing model achieved an area under the curve of 0.86 with driving features combined with total medication use. These findings suggest that naturalistic driving data holds high potential as a functional digital neurobehavioral marker for AI identifying depression in older adults on a national scale, thereby ensuring equitable access to treatment.

## Introduction

In 2022, depression prevalence in the United States (US) was 13%, with a resulting economic burden estimated at $233 billion (2016 dollars) and higher rates observed among women compared to men^1,2^. Major Depressive Disorder (MDD) affects 14.4% of older adults in the US, with 5.4% (2.2 million) having an active diagnosis of MDD, while 9.0% (3.7 million) had a history of MDD^3^. Globally, a meta-analysis of 48 studies found that ~28% of older adults are affected by depression^4^. MDD often goes undiagnosed and untreated and affects 6% to 9% of older adult patients in primary care clinics but increases to 12% to 14% among nursing home residents^5^. Depression can mimic cognitive decline via symptoms such as memory impairment, difficulty concentrating, and slowed thinking—core clinical phenotypes that also characterize mild cognitive impairment (MCI) and early Alzheimer’s disease (AD). Conversely, MCI and early AD may present with mood changes, such as apathy or withdrawal, that are often mistaken for depressive disorders. This diagnostic ambiguity can lead to delayed or inappropriate treatment, exacerbating patient outcomes. A meta-analysis study reported around 32% of individuals diagnosed with MCI also experience depressive symptoms^6^. Early and precise diagnosis is critical to ensuring patients receive the most effective, tailored interventions for their condition.

MDD is associated with poorer driving performance, slower steering reaction times, and a higher risk of crashes^7^. Older drivers, particularly those over age 65, are more likely to be involved in crashes, and this risk is expected to rise as the older population grows^8,9^. Increased crash risk factors include decreased visual function, slower reaction times, attention deficits, and memory impairments worsened by medication side effects and conditions like dementia and depression^10–12^. Antidepressants can influence driving safety via side effects such as drowsiness^12^. Older adults with depression are three times more likely to receive a marginal or fail rating on a standardized road test than older adults without depression over a two-and-a-half-year follow-up period^13^. A recent case-control study examined naturalistic driving behavior in 85 older adults with MDD (mean age, 69 years; 71% women) and 310 older adults without MDD (mean age, 70 years; 49% women) with a mean of 1.1 years of follow-up driving data^14^. Older adults with MDD showed significantly more hard braking and hard cornering events per trip than those without MDD. They traveled farther from home, visited more unique destinations, and exhibited increased entropy in driving patterns, indicating less predictable routes than those without MDD^14^.

Hence, subtle changes in behind-the-wheel performance across time could help identify depression earlier in this age group, thereby ensuring appropriate treatments are provided and preventing adverse outcomes. Current measures used to screen for depression among older adults lack specificity and sensitivity due to overlapping symptoms with other common diagnoses, like MCI, AD, or even medication side effects, which can mask or mimic depression. Older adults are also less likely to report emotional distress and reach out to mental health treatment due to stigma, generational attitudes toward mental health, or confusion as to whether depressive symptoms like fatigue or sleep disturbances are typical aspects of aging^15^.

Across all age groups, driving a personal vehicle is integral for independence, life satisfaction, and maintaining social connections. In the US, driving is the preferred mode of personal transportation due, in part, to a lack of viable public transit options^16^. As more older adults continue working beyond their 70 s, maintaining the ability to drive is essential for commuting and other daily activities^17^. Despite increasing alternatives, many older adults prefer driving themselves using personal vehicles^18^. This reliance on personal vehicles is reflected in vehicle ownership, with today’s young adults owning fewer vehicles compared to previous generations at the same age^19^. In 2050, the older adult population (age ≥ 65 years) will reach 88 million, representing 25% of all licensed drivers in the US^9,20^.

Naturalistic driving captured using in-vehicle data can predict older adults at a higher risk for conditions like MCI and preclinical AD^21^. The research objective of this study is to determine if naturalistic driving behavior captured via in-vehicle GPS dataloggers can identify depression in older adults using machine learning. We hypothesized that driving behaviors can distinguish between depressed and non-depressed older drivers using machine learning algorithms with 80% accuracy. Additionally, we hypothesized that integrating demographic variables and medication use would improve the model’s discriminatory performance.

## Results

### Participant characteristics

The study included 157 recruited participants, 81 classified as depressed and 76 as non-depressed (Table 1). The average age of participants was 72.93 years (standard deviation [SD] = 5.36), with a statistically significant difference between the depressed and non-depressed groups. Depressed participants were younger, with a mean age of 70.2 years (SD = 4.0), compared to 75.84 years (SD = 5.09) in the non-depressed group (*p* < 0.001). Sex was significantly different between the two groups (*p* < 0.001), with a higher proportion of females with depression (70.37% vs. 34.21%). The overall mean years of education was 16.59 years, and there was no difference between those with and without depression. Participants with depression reported taking a higher number of medications (mean = 0.52, SD = 0.85) than non-depressed (mean = 0.20, SD = 0.59). For those who endorsed antidepressant use, 13.58% of the MDD group reported using antidepressants, compared to 2.63% in the non-depressed group (*p* = 0.028).

**Table 1:** Descriptive analysis of demographics, medication usage, and monthly aggregated driving features used in models

	Overall (*n* = 157)	Non-depressed (*n* = 76)		Depressed (*n* = 81)	*p* value (chi-square/t-test)
Age, years	72.93 ± 5.36*	75.84 ± 5.09	70.20 ± 4.00		<0.001*
Sex (Female), *n* (%)	83 (52.87)*	26 (34.21)	57 (70.37)		<0.001*
Education, years	16.59 ± 2.36	16.87 ± 2.22	16.33 ± 2.47		0.156
Total classes of Medication	0.36 ± 0.75*	0.20 ± 0.59	0.52 ± 0.85		0.007*
Antidepressants, *n* (%)	13 (8.28)*	2 (2.63)	11 (13.58)		0.028*
Rate of Hardcore Braking Events	0.0010 ± 0.0025*	0.0008 ± 0.0020	0.0011 ± 0.0029		<0.001*
Rate of Hard cornering Events	0.0241 ± 0.0521*	0.0323 ± 0.0503	0.0155 ± 0.0525		<0.001*
Number of Days Driven	20.81 ± 7.69	20.73 ± 7.66	20.89 ± 7.72		0.568
Number of Trips less than 1 mile	18.56 ± 16.48*	19.34 ± 17.09	17.75 ± 15.78		0.005*
Number of Trips between 1 mile to 5 miles	42.40 ± 31.53*	41.04 ± 28.19	43.83 ± 34.62		0.011*
Random Entropy	5.02 ± 0.96	5.03 ± 0.94	5.01 ± 0.98		0.485
Radius of Gyration	73.55 ± 268.48	76.69 ± 270.33	70.26 ± 266.57		0.490
Maximum Distance from Home	409.20 ± 1706.26	427.14 ± 1755.02	390.46 ± 1654.18		0.535
Maximum of Distance	58.72 ± 69.92	56.71 ± 67.81	60.82 ± 72.02		0.091
Number of Unique Destinations	38.68 ± 21.38	38.59 ± 19.94	38.76 ± 22.78		0.821
Fall	843 (25.3)	441 (25.9)	402 (24.7)		0.447
Spring	764 (22.9)	375 (22.1)	389 (23.9)		
Summer	842 (25.3)	442 (26.0)	400 (24.6)		
Winter	880 (26.4)	442 (26.0)	438 (26.9)		

*Results indicate statistical significance at an error rate of 5%.

Descriptive statistics of driving features are also presented in Table 1. The number of hardcore braking events was statistically higher in the depressed group compared to the non-depressed group. In contrast, non-depressed participants had more hard cornering events (mean = 0.032, SD = 0.050) than their depressed counterparts (mean = 0.016, SD = 0.053, *p* < 0.001). Participants in the non-depressed group made more trips of less than one mile (mean = 17.75, SD = 15.78) compared to the depressed group (mean = 19.34, SD = 17.09, *p* = 0.005), while the depressed group reported more trips between 1 and 5 miles (mean = 43.83, SD = 34.62) than the non-depressed group (mean = 41.04, SD = 28.19, *p* = 0.011). No statistically significant differences were observed between the two groups regarding diving features: days driven per month, random entropy, radius of gyration, maximum distance from home, maximum distance traveled, and the number of unique destinations (Table 1). Descriptive characteristics for the train and test sets are reported in Supplementary Table 1 for all model input variables. Differences in the CDR between the groups and distribution of data collection durations between the depression and non-depression groups were observed (Supplementary Table 2) and visualized in Supplementary Fig. 1. Participants with MDD were more likely to have a higher CDR Sum of Boxes and a slightly lower number of months of driving data compared to those without MDD. There were no differences in the Area Deprivation Index that examined the socioeconomic condition of neighborhood residence between the two groups.

### Model performance

**Fig. 1:**
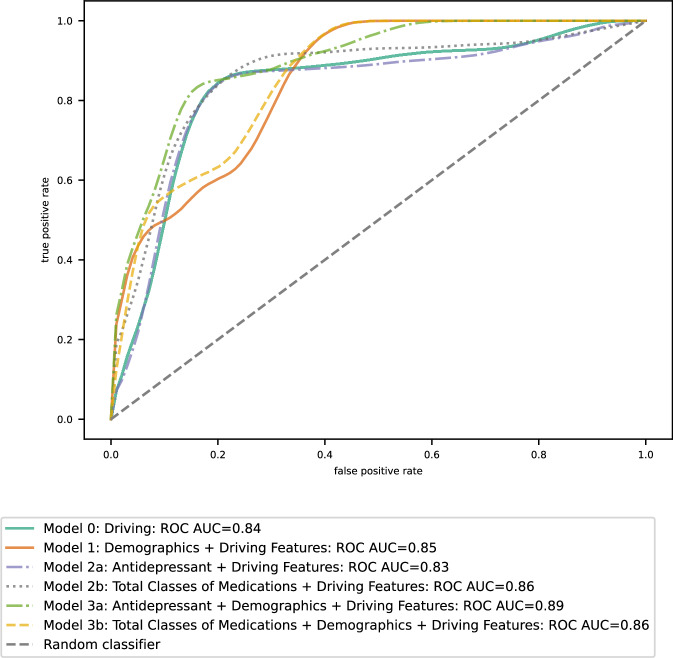
The final area under the receiver operating curves (AUC) for each model. AUC with 95% confidence interval was calculated using bootstrapped subsamples (1000 iterations with a 25% test set).

**Fig. 2:**
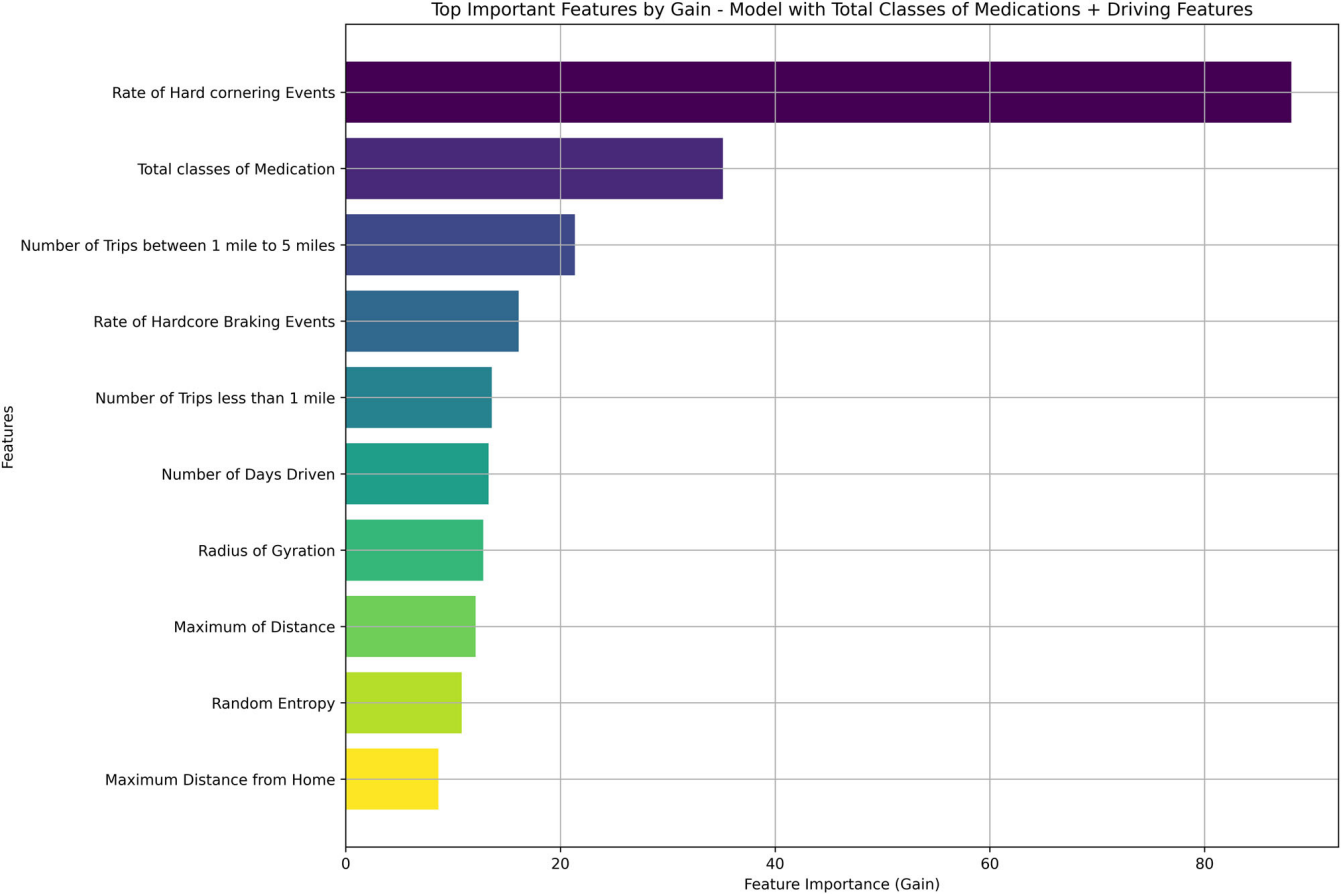
Feature importance ranking. Feature importance was calculated using the Gain metric for the total classes of medication and driving features model.

The precision, recall, F1, AUC, and specificity scores for each model are presented in Table 2 and Supplementary Table 3. Driving features alone were used to identify depression status with an F1 score of 0.82 (95% confidence interval [CI], 0.77 to 0.87). Demographic features with driving features generated an F1 score of 0.79 (95% CI: 0.73 to 0.84). Additionally, total classes of medications were combined with driving features, resulting in an improved overall model performance. This model using total classes of medications and driving features achieved an F1 score of 0.81 (95% CI: 0.76 to 0.86), along with the model combining antidepressants and driving features with an F1 score of 0.81 (95% CI: 0.76 to 0.87). Adding demographic variables to the medication models yielded an F1 score of 0.78 (95% CI: 0.72 to 0.84) for the antidepressants model and 0.79 (95% CI: 0.74 to 0.84) for the total medications class model (Fig. 1). The five important features in the model that captured total medications class and driving features included rate of hard cornering, total medications class, number of trips between one mile to five miles, rate of hardcore braking events, and number of trips less than one mile (Fig. 2).

**Table 2:** Assessment of the model performance on the test set

Model inputs	Precision	SD	Recall	SD	F1	SD	AUC	SD	Rank
Driving features	0.79 (0.72–0.86)	0.04	0.86 (0.79–0.92)	0.03	0.82 (0.77–0.87)	0.03	0.84 (0.78–0.89)	0.03	2
Demographics and driving features	0.70 (0.62–0.76)	0.04	0.91 (0.86–0.96)	0.03	0.79 (0.73–0.84)	0.03	0.85 (0.80–0.89)	0.02	4
Antidepressants and driving features	0.79 (0.72–0.86)	0.04	0.84 (0.77–0.90)	0.03	0.81 (0.76–0.87)	0.03	0.83 (0.77–0.88)	0.03	5
**Total classes of medications and driving features**	**0.74 (0.67–0.81)**	**0.04**	**0.90 (0.84–0.95)**	**0.03**	**0.81 (0.76–0.86)**	**0.03**	**0.86 (0.81–0.91)**	**0.02**	**1**
Antidepressants, demographics, and driving features	0.70 (0.63–0.78)	0.04	0.88 (0.82–0.94)	0.03	0.78 (0.72–0.84)	0.03	0.89 (0.86–0.93)	0.02	5
Total classes of medications, demographics, and driving features	0.70 (0.63–0.77)	0.04	0.92 (0.86–0.96)	0.03	0.79 (0.74–0.84)	0.03	0.86 (0.81–0.90)	0.02	3
Logistic Regression for demographics and antidepressants	0.67		0.59		0.63		0.80		7

Logistic regression was not evaluated by bootstrapping because the resampled data only contained one group. Bolded model performs the best.

Values in parentheses represent 95% confidence intervals after bootstrapping.

Finally, compared with XGBoost classifier, logistic regression using demographic variables to identify the outcome produced an F1 score of 0.63. Antidepressants alone produced an F1 score of 0.11, while the combination of both demographics and antidepressants resulted in an F1 of 0.62. Sensitivity and specificity evaluation metrics are provided in Supplementary Table 3 to provide additional model interpretability. The data aggregated at the weekly level was run with the same model without any significant differences from the monthly aggregation (See Supplementary Table 4). In an additional sensitivity analysis, after removing 13 participants who were using antidepressants, the model combining the total number of medications, demographics, and driving features ranks the best among all four models (See Supplementary Table 5).

## Discussion

Our findings suggest that naturalistic driving behaviors can differentiate between older adults with and without MDD. We rejected our first null hypothesis (no difference between MDD and non-MDD OA drivers) and accepted the alternate, as a single model using only driving features achieved a good F1 score (0.82), 80% specificity and high AUC (84%). The addition of total classes of medications and driving features further improved performance, achieving a high F1 score (0.81), comparable AUC (86%), precision (74%), and recall (90%). The higher recall indicates the model is particularly effective at identifying individuals who may be depressed. Among all six XGBoost models, the model with total classes of medications and driving features was ranked as the best-performing model, combining the four machine learning parameters. While the antidepressant and driving features model emerged as the fifth rank, it achieved a balance between sensitivity (84%) and specificity (81%). This balance is particularly important in clinical settings, as it minimizes both false negatives and false positives.

We could not fully reject our second null hypothesis (the addition of both demographic and medication features does not improve the model). When adding only medications, the model's accuracy improved. However, the addition of demographic variables (age, sex, years of education), alone or in combination with medications like antidepressants, resulted in a comparatively less accurate model, particularly in terms of specificity and sensitivity. Overall evaluation metrics indicated that models with demographic variables were overfitted, while other XGBoost models did not. This suggests that demographic variables have led to models that generalized poorly to unseen data. Compared to the low precision values in Table 2, these results likely indicate that a larger-than-expected proportion of the predicted depression cases were false positives. The false positives could be the result of the potentially collinear age and sex demographic variables seen in Table 1, indicating the models incorporate demographic variables incorrectly flagged non-depressed, younger, and/or female individuals as depressed.

Previous studies identified demographic factors such as older age and being female as risk factors for depression, and higher levels of education are often considered protective factors^22–24^. However, the logistic regression showed that demographics had a below-average F1 score (0.63). This finding suggests that only using demographic variables (age, sex, and years of education) provided limited predictive power for depression in older adults, emphasizing that depression in older adults is a complex issue influenced by more than just those three demographic factors. Other factors such as race, income, physical illness, and loneliness, may play significant roles in depression in this population^24–26^. Additionally, the high prevalence of comorbid medical conditions among older adults can obscure or mimic the symptoms of depression. Somatic complaints such as fatigue, weight loss, and sleep disturbances, are common in both depression and chronic illnesses, making differential diagnosis difficult. This overlap often leads to underdiagnosis or misdiagnosis of depressive disorders, where clinicians may attribute symptoms to aging or physical health issues rather than mental health concerns.

The discriminatory power of driving features alone aligns with earlier findings and supports previous findings that the differences in driving behaviors, a complex instrumental daily living activity, can predict conditions such as preclinical AD^27^. Depression in older adults often manifests through slower reaction times, decreased attentional control, and diminished decision-making capacity^7^. These impairments may manifest in the observed hard braking, hard cornering, and longer trips. Screening for depression in clinical healthcare settings among older adults is of critical importance due to the growth of the aging population, the high prevalence of depressive symptoms in this population, and the significant impact of untreated depression on overall health and well-being. Depression in older adults is often underdiagnosed and undertreated, as symptoms may be mistaken for normal aging processes or overlooked in the presence of coexisting medical conditions. Furthermore, antidepressants are known to impact cognitive and driving ability, which could explain why their inclusion enhanced the model’s performance^12^.

Detection of depression among older adults presents several challenges, including time constraints, inconsistent use of validated screening tools, and the variability of patient disclosure due to stigma or discomfort in discussing mental health issues. Traditional screening methods, such as self-report questionnaires or clinician-administered interviews, can be limited by subjective interpretation and are often influenced by the clinician’s time availability and the patient’s willingness to engage. Additionally, the subjective nature of depressive symptoms complicates accurate diagnosis, as they often overlap with other medical conditions. However, advancements in technology, including artificial intelligence (AI), machine learning algorithms, and digital health tools, offer the potential to enhance the screening process by improving precision and efficiency. AI-driven tools can analyze large sets of patient data, including behavioral and physiological indicators, to identify patterns indicative of depression, thereby facilitating early detection. Digital platforms may also deliver standardized and validated screening tools at scale, reducing variability and improving accessibility, allowing for more frequent and accurate assessments in diverse patient populations.

This study has several strengths. First, it used real-world, naturalistic driving data, offering valuable insights into participants’ daily lives and providing a practical tool for future research and screening. Second, it employed a rigorous data analysis approach. It applied GridSearchCV, combined with 10-fold GroupKFold cross-validation, to find the best hyperparameters of the models. This method identified an optimal combination of hyperparameters that maximized model performance. Recall scores in GridSearchCV were prioritized to ensure that the model captured as many true cases of depression as possible. Finally, our study introduces a new approach to passively identifying and screening for depression in older populations. In addition to these strengths, the study offers promising clinical implications for real-world applications. Driving data can be collected through simple onboard diagnostic devices and can be paired with wearables like a smartwatch to capture real-time physiological data, such as respiratory rate, heart rate, or stress levels, which can provide early detection of health conditions like depression screening, making it more accessible and cost-effective. This approach leverages the high vehicle ownership rates in countries like the US to monitor depression among most drivers passively. Furthermore, dataloggers could contribute to public health efforts by aggregating anonymized health data to track the prevalence of certain conditions within populations, thereby informing healthcare resource allocation and regional disease surveillance. However, we acknowledge that individuals without personal vehicles or adequate insurance coverage may not benefit from this type of screening.

Our study has a few limitations. While participants who shared vehicles with others were excluded, there remains the possibility that friends, relatives, or other household members may have contributed to driving data without our knowledge. However, with the large volume of trips collected and then aggregated monthly, potential noise introduced into the dataset is anticipated to be minimized. Second, reliance on self-reported medication use may not be accurate or up-to-date, and changes in medication over time were not tracked. This may impact our findings, particularly for acute conditions requiring shorter treatment between visits. Since certain medications may be prescribed for acute conditions (e.g., pain) and shorter durations, the performance of our models could be affected. Since this was a prospective cohort study, detailed electronic health record data (EHR) on our participants’ psychiatric history or the specific purpose of antidepressant use were not available, which may introduce a confounding variable that affects driving behavior. Additionally, there was a lack of data on over-the-counter medications, such as sedative antihistamines, which may affect driving performance. Future research should include EHR data and other medications to adroitly tease apart their individual contributions to understand their impact on driving behavior better. Additionally, older adults may exhibit atypical symptoms of depression, such as cognitive impairment, fatigue, or somatic complaints, rather than the mood disturbances typically associated with the condition, complicating the diagnostic process. Given the focus on older adults, some participants may have experienced cognitive decline or preclinical AD, factors that were not controlled for in this study but which could have influenced both their driving behavior and depressive symptoms. Machine learning algorithms could identify subtle, condition-specific markers by analyzing detailed driving patterns—such as reaction to traffic signals, lane-keeping, braking behaviors, and speed variability—alongside supplementary data like physiological metrics or cognitive assessments. Given the symptom overlap between AD and MDD, AD-related changes may exhibit more pronounced spatial navigation errors, while MDD might show more irregularities linked to psychomotor slowing or hesitation. A robust classification model, trained on diverse datasets, could improve diagnostic accuracy and provide an early, non-invasive screening tool, contributing to safer roads and timely, targeted interventions for individuals with these conditions. Furthermore, participants in the depressed group were younger and more likely to be female and more likely to be prescribed antidepressants, which may limit the generalizability of our findings. To address these issues, passive real-world driving data captured included only significant variables, recruited balanced groups of depressed and non-depressed participants, encoded categorical variables, and tuned XGBoost models using parameters such as gamma, learning_rate, max_depth, subsample, and colsample_bytree, along with cross-validation^28^.

In conclusion, this study provides a promising approach for future research into objective and cost-effective methods for identifying depression in older adults. This could not only improve mental health outcomes but also enhance driving safety by reducing the risk of crashes related to depression. Future research should consider recruiting a larger sample size and extending the observation period to strengthen the robustness of the findings by utilizing more sophisticated modeling techniques, such as deep learning algorithms, to capture more complex patterns. Developing more sophisticated techniques to distinguish between drivers could help ensure that all trips analyzed come from the intended participant, improving data quality. Incorporating additional contextual data, such as cognitive assessments or health records, may also help unravel the effects of cognitive impairment or preclinical AD on driving behaviors and depression.

## Methods

### Recruited participants

Participants were enrolled in longitudinal studies of aging, driving, and depression (R01AG068183, R01AG067428) at Washington University School of Medicine^29^. To be eligible, participants had to: (1) be age 65 years or older at baseline, (2) be cognitively normal based on a 0 on the Clinical Dementia Rating (CDR), (3) have a valid driver’s license, (4) drive at least once weekly, and (5) complete annual clinical and neuropsychological assessments. Participants provided written informed consent, and all study procedures were approved (202010214, 202003209) by the Washington University Human Research Protection Office.

### Naturalistic driving

The *Driving Real-world In-Vehicle Evaluation System* (DRIVES) methodology, which has been previously published^30–34^, captured continuous and objective data on driving behaviors from the personal vehicles of older adults. A commercial GPS datalogger (G2 Tracking Device™, Azuga Inc, San Jose, CA) collected driving behaviors using a GPS, accelerometer, and gyroscope. Powered by the vehicle’s battery, it is plugged into its OBDII port, and data are transmitted every 30 s from ignition on to off. A trip summary report included date and time stamp, GPS location, vehicle speed, and accelerometer-triggered events like speeding, hard braking, and sudden acceleration.

Data were extracted between July 1, 2022, and July 1, 2024, capturing monthly and seasonal driving behavior changes. Each participant in the study had a minimum of 10 months of driving data, ensuring the majority of the year was captured, accounting for medical surgery procedures or relocating to the south during winter months. Supplementary Table 6 details the driving behaviors measured using ten variables. These variables were aggregated monthly to capture recruited participants’ driving behaviors and were selected based on preliminary analyses, and only variables shown to be significant were included in the study^14^. Seasons were incorporated into the analysis using a four-season variable (winter [December, January, February], spring [March, April, May], summer [June, July, August], and fall [September, October, November]) to capture seasonal effects on driving behaviors. Participants who reported sharing their vehicles with other drivers were excluded from the analysis. Each month’s record for each participant was considered a data point.

### Depression

Recruited participants were classified as either depressed or non-depressed. Depression status was determined using a MDD diagnosis by a clinician and confirmed by a Patient Health Questionnaire-9 (PHQ-9) score of 10 or higher, which corresponds to moderate to severe depression^35^. The non-depressed group was defined by self-report, indicating no MDD, and a PHQ-9 score of 0, indicating no depressive symptoms. Depression status was selected at their annual visit closest to July 1, 2022, to avoid further variance in the analysis. Depression status was treated as a binary variable, with depressed encoded as one and non-depressed encoded as zero.

### Demographic and clinical data

Demographic data, including age, sex, and years of education, were self-reported. Medication data were collected at annual office visits. Participants confirmed medications at home and then self-reported the use of all medications and supplements taken within the past year. Data from recruited participants’ visits close to July 1, 2022 were used. Medications were classified based on the American Hospital Formulary Service classification system^36^. Antidepressants were coded as a binary variable (taken or not taken), and the total number of medication classes was captured as a continuous variable. A comprehensive list of all participant medications is in Supplementary Table 7.

### Statistical analysis

Extreme Gradient Boosting (XGBoost) was used since it is an ensemble learning algorithm that minimizes loss using second-order gradients and advanced regularization (L1 and L2), which reduces overfitting and improves model generalization and performance^37,38^. XGBoost is known for its ability to handle outliers, scalability, speed, and accuracy in large datasets^38^. It is also highly customizable through hyperparameter tuning, enabling optimized performance. Logistic regressions, incorporating demographic variables (age, sex, years of education), antidepressants, and a combination of both, were also used to establish baselines for identifying depression status.

Six models were trained with XGBoost on different sets: (1) driving features (Supplementary Table 6), (2) demographic (age, sex, years of education) and driving features, (3) antidepressant use and driving features, (4) total classes of medications used and driving features, (5) antidepressant use, demographic and driving features, (6) total classes of medication used, demographic and driving features. Data was split into 70% training and 30% test sets, with monthly aggregated data for each participant assigned exclusively to either training or test sets to avoid target leakage between the sets and eliminate overfitting.

**Fig. 3:**
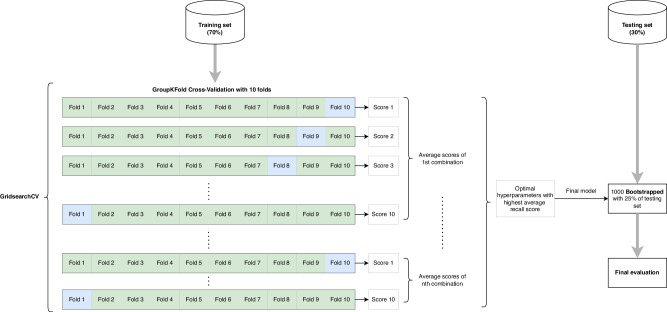
The workflow of the XGBoost classifier includes hyperparameter turning, 10-Fold GroupKFold cross-validation, and bootstrapped test set validation. The figure displays the methodological workflow described in the paper.

OneHotEncoder encoded categorical variables. Hyperparameter tuning was conducted using GridSearchCV, which performs an exhaustive search of specified hyperparameter values^39^. Integrated with the 10-fold GroupKFold cross-validation on the train set, this approach identified the optimal combination of hyperparameters that maximized model performance, focusing on optimizing recall. The hyperparameters explored included the learning rate, maximum depth, gamma, and subsample. The best-performing hyperparameters were then applied to train the final model (Fig. 3). Overfitting was examined by testing all trained models on the training set and comparing their metrics with those from the test set.

Model performance was evaluated on bootstrapped subsamples (1000 iterations with a 25% test set) of the test set to determine the precision, recall, F1 score, and Area Under the Receiver Operating Characteristic (AUC) score with 95% confidence intervals. Specifically, in each iteration, a bootstrapped sample was drawn from the test set, which consisted of 25% of the test data. Each model was ranked individually based on Precision, Recall, F1, and AUC scores, and the average of these ranks was then used to calculate an overall performance ranking. The hyperparameters used in the grid search and their corresponding ranges of values are available in Supplementary Table 8. All analyses were performed in Python version 3.11.5 on Jupyter Lab, utilizing TableOne 0.9.1, Scikit-learn 1.5.1, and XGBoost 2.1.1^38–40^.

## Supplementary information

Supplementary information

## Data Availability

The raw data supporting the conclusions of this article and additional related documents are available from the corresponding author on reasonable request.

## Code availability

The code used has been made available on GitHub (https://github.com/cchen-bit/Depression-Driving-and-ML‘) for interpretation and reproducibility.
