# Supplementary material for: Identifying major depressive disorder in older adults through naturalistic driving behaviors and machine learning
Source: NPJ Digit Med. 2025 Feb 15;8:102. doi: 10.1038/s41746-025-01500-w (PMC11828977; doi:10.1038/s41746-025-01500-w)
Supplement: Supplementary file 1 — Supplementary information [file 41746_2025_1500_MOESM1_ESM.docx]

**Supplementary Figure 1. Distribution of Data Collection Duration Across Depressed and Non-Depressed Groups**
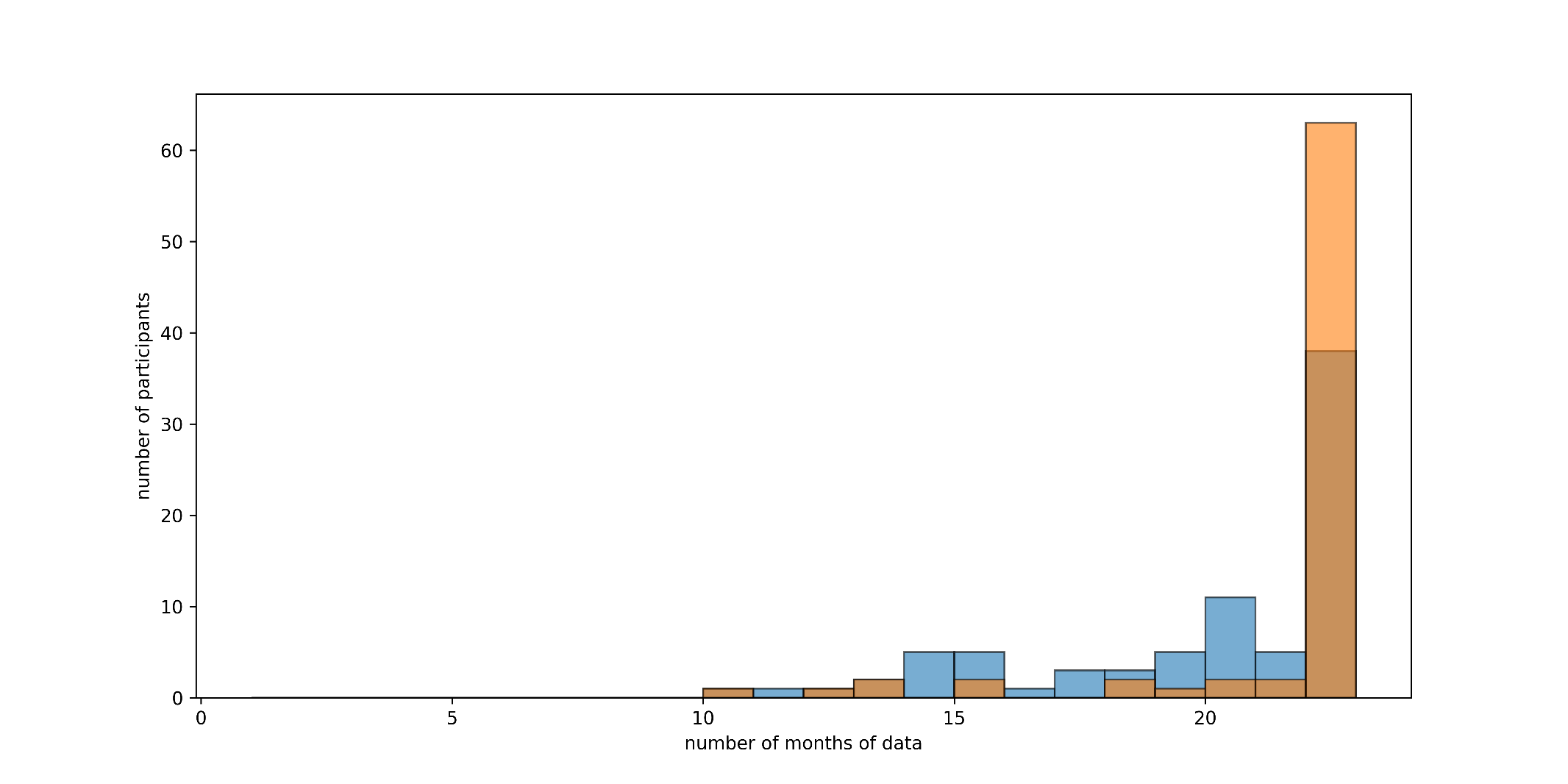


The figure displays the distribution of data collection duration for participants in the depressed (blue) and non-depressed (yellow) groups. All study participants have more than 10 months of data within the study period.

**Supplementary Table 1. Descriptive Analysis of Input features for Train and Test Set and p-values.**

|  |  | **Train (n = 2325)** | **Test (n = 1004)** | ***p* value**  **(chi-square/t-test)** |
| --- | --- | --- | --- | --- |
| Depression, n (%) | Depressed | 1161 (49.9) | 468 (46.6) | 0.085 |
|  | Non-depressed | 1164 (50.1) | 536 (53.4) |  |
| Age |  | 73.58 (5.71) | 72.49 (4.52) | <0.001* |
| Sex, n (%) | Male | 1176 (50.58) | 419 (41.73) | <0.001* |
|  | Female | 1149 (49.42) | 585 (58.27) |  |
| Antidepressant use, n (%) | Yes | 204 (8.77) | 55 (5.48) | 0.001* |
|  | No | 2121 (91.23) | 949 (94.52) |  |
| Years of education |  | 16.73 (2.38) | 16.44 (2.30) | 0.001* |
| Total classes of Medication |  | 0.38 (0.77) | 0.31 (0.70) | 0.013* |
| Rate of Hardcore Braking Events |  | 0.001 (0.002) | 0.001 (0.003) | 0.012* |
| Rate of Hard cornering Events |  | 0.022 (0.051) | 0.028 (0.054) | 0.004* |
| Number of Days Driven |  | 20.44 (7.69) | 21.66 (7.60) | <0.001* |
| Number of Trips less than 1 mile |  | 18.27 (16.37) | 19.24 (16.73) | 0.124 |
| Number of Trips between 1 mile to 5 miles |  | 41.61 (31.80) | 44.24 (30.83) | 0.025* |
| Random Entropy |  | 4.95 (0.94) | 5.19 (0.98) | <0.001* |
| Radius of Gyration |  | 72.69 (279.50) | 75.53 (241.15) | 0.767 |
| Maximum Distance from Home |  | 394.00 (1680.81) | 444.38 (1764.13) | 0.443 |
| Maximum of Distance |  | 54.88 (68.33) | 67.62 (72.74) | <0.001 |
| Number of Unique Destinations |  | 36.50 (19.35) | 43.71 (24.74) | <0.001 |
| Fall |  | 590 (25.4) | 253 (25.2) | 0.949 |
| Spring |  | 534 (23.0) | 230 (22.9) | 1.00 |
| Summer |  | 589 (25.3) | 253 (25.2) | 0.97 |
| Winter |  | 612 (26.3) | 268 (26.7) | 0.86 |

*Note.* *Results indicate statistical significance at an error rate of 5%.

**Supplementary Table 2. Distribution of CDR, ADI Scores, and Number of Months Across Depressed and Non-Depressed Groups**

|  |  | **Overall**  **(n = 157)** | **Non-depressed**  **(n = 76)** | **Depressed**  **(n = 81)** | **p-Value** |
| --- | --- | --- | --- | --- | --- |
| CDR sum of box, n (%) |  | 0.63 (0.85) | 0.37 (0.84) | 0.88 (0.79) | <0.001*** |
| CDR, n (%) | **0.0** | 99 (63.1)*** | 61 (80.3) | 38 (46.9) | <0.001*** |
|  | **0.5** | 57 (36.3)*** | 14 (18.4) | 43 (53.1) |  |
|  | **1.0** | 1 (0.6)*** | 1 (1.3) |  |  |
| ADI National Rank, mean ±SD |  | 45.3 ±24.9 | 44.9 ±24.6 | 45.7 ±25.2 | 0.834 |
| Number of Months |  | 20.8 (3.4)*** | 21.8 (2.8) | 19.9 (3.5) | <0.001*** |

Note: CDR (Clinical Dementia Rating); ADI (Area Deprivation Index).

*Results indicate statistical significance at an error rate of 5%.

**Supplementary Table 3. Results of XGBoost Models with Sensitivity and Specificity**

| **Model inputs** | **Sensitivity (Recall)** | **SD** | **Specificity** | **SD** |  |
| --- | --- | --- | --- | --- | --- |
| Driving features | 0.86 (0.79–0.92) | 0.03 | 0.80 (0.73–0.87) | 0.03 |  |
| Demographics and driving features | 0.91 (0.86–0.96) | 0.03 | 0.65 (0.57–0.73) | 0.04 |  |
| Antidepressants and driving features | 0.84 (0.77–0.90) | 0.03 | 0.81 (0.74–0.87) | 0.03 |  |
| Total classes of medications and driving features | 0.90 (0.84–0.95) | 0.03 | 0.73 (0.65–0.80) | 0.04 |  |
| Antidepressants, demographics and driving features | 0.88 (0.82–0.94) | 0.03 | 0.68 (0.60–0.76) | 0.04 |  |
| Total classes of medications, demographics and driving features | 0.92 (0.86–0.96) | 0.03 | 0.65 (0.57–0.74) | 0.04 |  |

**Supplementary Table 4. XGBoost Performance on Weekly-Level Aggregated Data**

| **Phase** | **Precision** | **Recall** | **F1 Score** | **ROC AUC** |
| --- | --- | --- | --- | --- |
| Driving Features | 0.72 | 0.90 | 0.80 | 0.82 |
| Demographics + Driving Features | 0.67 | 0.90 | 0.76 | 0.83 |
| Antidepressant + Driving Features | 0.72 | 0.91 | 0.81 | 0.84 |
| Total + Driving Features | 0.68 | 0.91 | 0.78 | 0.76 |
| Antidepressant + Demographics + Driving Features | 0.71 | 0.72 | 0.72 | 0.78 |
| Total + Demographics + Driving Features | 0.69 | 0.81 | 0.75 | 0.85 |

**Supplementary Table 5. XGBoost Performance on Participants without Antidepressants**

| **Phase** | **Precision** | **Recall** | **F1 Score** | **ROC AUC** | **Specificity** |
| --- | --- | --- | --- | --- | --- |
| Driving Features | 0.77 | 0.85 | 0.81 | 0.85 | 0.82 |
| Demographics + Driving Features | 0.82 | 0.77 | 0.79 | 0.92 | 0.88 |
| Total Medication Used + Driving Features | 0.71 | 0.88 | 0.79 | 0.84 | 0.75 |
| Total Medication Used + Demographics + Driving Features | 0.82 | 0.84 | 0.83 | 0.96 | 0.87 |

**Supplementary Table 6. Description of the GPS-based driving features.**

|  | **Indicator (aggregated monthly)** | **Units** | **Description** |
| --- | --- | --- | --- |
| 1 | Number of Hardcore Braking Events* | Count | Total number of detected events where a vehicle moving at a speed greater than 18.64 miles per hour (MPH) (30 kilometer per hour (KPH)) shows a decrease in speed exceeding a rate of 11.81 MPH (19 KPH) per second. |
| 2 | Rate of Hardcore Braking | Events per mile | Calculated by the total number of hardcore braking events divided by total distance (miles) traveled in each month. |
| 3 | Number of Hard Cornering Events* | Count | Total number of detected events where the lateral acceleration of the vehicle exceeds a threshold of 0.4 g-force (g), sustained for at least 12 counts (0.5s) within a sliding window size of 36 (1.5s). |
| 4 | Rate of Hard Cornering | Events per mile | Calculated by the total number of hard cornering events divided by total distance traveled in each month. |
| 5 | Number of Days Driven | Count | Total number of days per month when a vehicle completes a trip (ignition on / ignition off cycle) with a trip distance greater than 0 miles. |
| 6 | Number of Trips less than 1 mile | Count | Total number of trips with a distance smaller than 1 mile in each month. |
| 7 | Number of Trips between 1 mile to 5 miles | Count | Total number of trips with a distance between 1 and 5 miles in each month. |
| 8 | Random Entropy | Bits | $E_{rand}\left( u \right)={log}_{2} \left( N_{u} \right)$  $N_{u}$ is the number of distinct locations visited by $u$, capturing the degree of predictability of $u$’s whereabouts if each location is visited with equal probability^1^. |
| 9 | Radius of Gyration | Miles | Compute the radii of gyration of a set of individuals in a TrajDataFrame. The radius of gyration of an individual is defined as:  $r_{g}\left( u \right)= \sqrt{\frac{1}{n_{u}}\sum_{i=1}^{n_{u}} dist(r_{i}\left( u \right)-r_{cm}\left( u) \right)^{2}}$  where $r_{i}\left( u \right)$ represents the $n_{u}$positions recorded for $u$, and $r_{cm}(u)$ is the center of mass of $u$’s trajectory. In mobility analysis, the radius of gyration indicates the characteristic distance travelled by $u$^1^. |
| 10 | Maximum Distance from Home | Miles | Maximum distance travelled from home in each month.  Compute the maximum distance traveled from their home location by a set of individuals in a TrajDataFrame. The maximum distance from home $ⅆh_{\max}\left( u \right)$ of an individual is defined as:  $ⅆh_{\max}\left( u \right)= \max_{1\leqⅈ<j<n_{u}}dist(r_{i}, h\left( u \right))$  where $N_{u}$ is the number of points recorded for $u$, $r_{i}$is a location visited by $u$ described as a  pair, $h\left( u \right)$ is the home location of $u$, and $dist$ is the geographic distance between two points^1^. |
| 11 | Maximum of Distance | Miles | Maximum distance traveled in each month. |
| 12 | Number of unique destinations | Count | Total number of unique destinations visited in each month^1^. |
| 13 | Season |  | Winter (Dec, Jan, Feb), Spring (Mar, Apr, May), Summer (Jun, Jul, Aug), and Fall (Sep, Oct, Nov) |

*Variables were used to calculate the rate and were not directly included in the statistical analysis.

**Supplementary Table 7. Mapped medications* included in the analysis**

| **Predictive Class Label** | **Medication Class (if available)**  **Medication Name** |
| --- | --- |
| Antidepressants | ***Monoamine Oxidase Inhibitors (MAOI)***phenelzine, selegiline  ***Selective Serotonin Reuptake Inhibitors/ Serotonin And Norepinephrine Reuptake Inhibitors (SSRI/SNRI)***  citalopram, desvenlafaxine, duloxetine, escitalopram, fluoxetine, paroxetine, sertraline, venlafaxine, vilazodone  ***Tricyclic Antidepressants (TCAs)***  amitriptyline, doxepin, imipramine, nortriptyline |
| Total classes of medications | ***Anticonvulsants***  carbamazepine, clonazepam, gabapentin, lamotrigine, levetiracetam, pregabalin, primidone, topiramate |
|  | ***Antidepressants***  see above predictive class label |
|  | ***Antipsychotics***  aripiprazole, cariprazine, prochlorperazine, quetiapine |
|  | ***Benzodiazepine***  alprazolam, chlordiazepoxide, diazepam, lorazepam, temazepam, triazolam***Non-Benzodiazepine Sedative Hypnotics***  buspirone, eszopiclone, suvorexant, zaleplon, zolpidem |
|  | ***Central Nervous System (CNS) drugs***  acetaminophen, acetaminophen/acetylsalicylic acid/caffeine, acetaminophen/butalbital/caffeine, acetaminophen/caffeine, acetaminophen/codeine, acetaminophen/diphenhydramine, acetaminophen/hydrocodone, acetaminophen/oxycodone, acetaminophen/phenyltoloxamine, acetaminophen/propoxyphene, acetaminophen/pseudoephedrine, acetylsalicylic acid/anhydrous citric acid/sodium bicarbonate, acetylsalicylic acid/caffeine/propoxyphene, acetylsalicylic acid/dipyridamole, almotriptan, alprazolam, amitriptyline, amphetamine, aripiprazole, armodafinil, aspirin, atropine/hyoscyamine/phenobarbital/scopolamine, bupropion, buspirone, butalbital, carbamazepine, carbidopa/levodopa, cariprazine, celecoxib, chlordiazepoxide, citalopram, clonazepam, codeine/guaifenesin/pseudoephedrine, cyclohexane, desvenlafaxine, dextromethorphan/quinidine, diazepam, diclofenac, doxepin, duloxetine, eletriptan, escitalopram, eszopiclone, etodolac, fluoxetine, gabapentin, hydrocodone, ibuprofen, imipramine, indomethacin, ketorolac, lamotrigine, levetiracetam, lidocaine, lisdexamfetamine, lorazepam, meloxicam, memantine, methylphenidate, milnacipran, mirtazapine, modafinil, morphine, nabumetone, naltrexone, naproxen, nefazadone, nortriptyline, oxycodone, paroxetine, phenelzine, phentermine, piroxicam, pramipexole, pregabalin, primidone, prochlorperazine, procyclidine, propoxyphene, quetiapine, rofecoxib, ropinirole, secobarbital, selegiline, sertraline, sumatriptan, suvorexant, temazepam, topiramate, tramadol, trazodone, triazolam, tryptophan, venlafaxine, vilazodone, zaleplon, zolpidem, zolmitriptan |
|  | ***Opioids***  acetaminophen/codeine, acetaminophen/hydrocodone, acetaminophen/oxycodone, acetaminophen/propoxyphene, acetylsalicylic acid/caffeine/propoxyphene, codeine/guaifenesin/pseudoephedrine, hydrocodone, morphine, oxycodone, propoxyphene, tramadol |
|  | ***Non-Steroidal Anti-Inflammatory Drug(NSAID) and Acetaminophen(APAP)***  acetaminophen, acetaminophen/acetylsalicylic acid/caffeine, acetaminophen/butalbital/caffeine, acetaminophen/caffeine, acetaminophen/codeine, acetaminophen/diphenhydramine, acetaminophen/hydrocodone, acetaminophen/phenyltoloxamine, acetaminophen/propoxyphene, acetaminophen/pseudoephedrine, acetylsalicylic acid/anhydrous citric acid/sodium bicarbonate, acetylsalicylic acid/caffeine/propoxyphene, acetylsalicylic acid/dipyridamole, aspirin, celecoxib, diclofenac, etodolac, ibuprofen, indomethacin, ketorolac, meloxicam, nabumetone, naproxen, piroxicam, rofecoxib |

*Only medications endorsed by study participants and mapped to this list were included in the analysis. All brand names are converted to generic names.

**Supplementary Table 8. List of Hyperparameters used in the Grid Search for XGBoost**

| **Parameter** | **Values** |
| --- | --- |
| n_estimators | [100, 200, 300] |
| learning_rate | [0.01, 0.1, 0.15, 0.3] |
| max_depth | [3, 4, 5, 6] |
| subsample | [0.6, 0.8, 1.0] |
| colsample_bytree | [0.8, 0.9, 1.0] |
| gamma | [0, 0.1, 0.2] |

**Supplementary References**

1. Pappalardo, L., Simini, F., Barlacchi, G. & Pellungrini, R. Scikit-mobility: A *python* library for the analysis, generation, and risk assessment of mobility data. *J. Stat. Softw.* **103**, 1–38 (2022). https://doi.org/10.18637/jss.v103.i04
